# Characterization of β-lactamase and virulence genes in *Pseudomonas aeruginosa* isolated from clinical, environmental and poultry sources in Bangladesh

**DOI:** 10.1371/journal.pone.0296542

**Published:** 2024-04-16

**Authors:** Raihana Islam, Farhana Binte Ferdous, M. Nazmul Hoque, Nowshad Atique Asif, Md. Liton Rana, Mahbubul Pratik Siddique, Md. Tanvir Rahman

**Affiliations:** 1 Department of Microbiology and Hygiene, Faculty of Veterinary Sciences, Bangladesh Agricultural University, Mymensingh, Bangladesh; 2 Department of Gynecology, Obstetrics and Reproductive Health, Faculty of Veterinary Medicine and Animal Science, Bangabandhu Sheikh Mujibur Rahman Agricultural University, Gazipur, Bangladesh; Abadan University of Medical Sciences, ISLAMIC REPUBLIC OF IRAN

## Abstract

The emergence and spread of multidrug-resistant pathogens like *Pseudomonas aeruginosa* are major concerns for public health worldwide. This study aimed to assess the prevalence of *P*. *aeruginosa* in clinical, environmental, and poultry sources in Bangladesh, along with their antibiotic susceptibility and the profiling of β-lactamase and virulence genes using standard molecular and microbiology techniques. We collected 110 samples from five different locations, *viz*., BAU residential area (BAURA; n = 15), BAU Healthcare Center (BAUHCC; n = 20), BAU Veterinary Teaching Hospital (BAUVTH; n = 22), Poultry Market (PM; n = 30) and Mymensingh Medical College Hospital (MCCH; n = 23). After overnight enrichment in nutrient broth, 89 probable *Pseudomonas* isolates (80.90%) were screened through selective culture, gram-staining and biochemical tests. Using genus- and species-specific PCR, we confirmed 22 isolates (20.0%) as *P*. *aeruginosa* from these samples. Antibiogram profiling revealed that 100.0% *P*. *aeruginosa* isolates (n = 22) were multidrug-resistant isolates, showing resistance against Doripenem, Penicillin, Ceftazidime, Cefepime, and Imipenem. Furthermore, resistance to aztreonam was observed in 95.45% isolates. However, *P*. *aeruginosa* isolates showed a varying degree of sensitivity against Amikacin, Gentamicin, and Ciprofloxacin. The *blaTEM* gene was detected in 86.0% isolates, while *blaCMY*, *blaSHV* and *blaOXA*, were detected in 27.0%, 18.0% and 5.0% of the *P*. *aeruginosa* isolates, respectively. The *algD* gene was detected in 32.0% isolates, whereas *lasB* and *exoA* genes were identified in 9.0% and 5.0% *P*. *aeruginosa* isolates. However, none of the *P*. *aeruginosa* isolates harbored *exoS* gene. Hence, this study provides valuable and novel insights on the resistance and virulence of circulating P. *aeruginosa* within the clinical, environmental, and poultry environments of Bangladesh. These findings are crucial for understanding the emergence of β-lactamase resistance in *P*. *aeruginosa*, highlighting its usefulness in the treatment and control of *P*. *aeruginosa* infections in both human and animal populations.

## 1. Introduction

*Pseudomonas aeruginosa*, a Gram-negative bacterium, has become increasingly recognized as an emerging opportunistic pathogen, leading to significant clinical implications, especially in hospital environments where it presents a substantial threat to immunocompromised individuals [1, 2]. *P*. *aeruginosa*, recognized for its versatility and metabolic variability, stands out as a significant factor in nosocomial infections, leading to severe acute and chronic illnesses in individuals with diverse vulnerabilities [3, 4]. Its ubiquitous presence is not limited to healthcare settings, as it thrives in a wide range of habitats, including soil, water, and even environments contaminated with oil, giving it the designation of a "ubiquitous" or "common soil and water bacterium." [5].

Antimicrobial resistance (AMR) in *P*. *aeruginosa*, is a significant concern in healthcare settings due to its ability to cause severe infections, particularly in individuals with weakened immune systems. *P*. *aeruginosa* displays intrinsic resistance to numerous antibiotics, and over time, it can further develop resistance mechanisms through genetic changes [6]. The problem of AMR in *P*. *aeruginosa* is a worrisome issue in the healthcare settings. The ability of this pathogen to acquire novel resistance traits adds further difficulties in managing and controlling associated infections. This situation has been characterized as a "crisis of the twenty-first century" [6, 7]. Antibiotic overuse has developed multidrug resistance (MDR) among bacteria, such as *P*. *aeruginosa*, posing a severe danger to public health and economies, particularly in low and middle-income countries (LMICs) in Africa and Asia, including Bangladesh. The increase in antibiotic-resistant strains of P. aeruginosa, where more than 10% of global isolates exhibit MDR, further compounds the challenges associated with treating infections caused by this bacterium [8]. Notably, *P*. *aeruginosa* uses a variety of resistance mechanisms, including gene expression under stress and the production of antibiotic-resistant biofilms, which reduces the efficiency of traditional therapies [9]. Antibiotic-resistant *P*. *aeruginosa* is present in diverse environmental settings, including wastewater and soils. This underscores the importance of investigating the frequency and resistance patterns of these clinically important bacteria in different contexts [10]. The recent widespread emergence of extended-spectrum beta-lactamase (ESBL)-producing *P*. *aeruginosa* poses a significant and alarming public health threat [11]. Moreover, the rising occurrence of ESBL-producing *P*. *aeruginosa* is consistently documented as a primary contributor to healthcare-associated infections in different countries of the globe [12, 13]. As a result of the complications imposed by ESBL-producing isolates, microbiologists, medics, and scientists working on creating novel antimicrobial medications face extra hurdles [11, 14].

*P*. *aeruginosa* is recognized for its extensive range of virulence factors, which play a substantial role in its pathogenicity and capacity to induce infections. Various virulence factors have been identified in *P*. *aeruginosa*, markedly influencing the bacterium’s pathogenic potential [4, 15]. Toxin A (*toxA*), exotoxin A (*exoA*), alkaline protease (*aprA*), elastase, and exoenzymes (S, U, and T *exoS*, *exoU*, *exoT*) are the principal virulence factors. *LasB*, a zinc metalloprotease, exhibits elastolytic activity specifically on lung tissue of human [16]. Chronic lung infections are significantly influenced by the *algD* gene, which is accountable for the alginate capsule of *P*. *aeruginosa* [17]. In addition, exotoxin A is secreted via Type II secretion systems, thereby contributing to the extracellular pathogenicity of the bacterium [18]. The existence and intricate interactions of these virulence factors in *P*. *aeruginosa* play a pivotal role in its pathogenicity, allowing it to instigate a diverse array of infections, especially in individuals with compromised immune systems or underlying health issues. A comprehensive understanding of these virulence mechanisms is essential for formulating targeted strategies to address *P*. *aeruginosa* infections and enhance patient outcomes [4].

While most *P*. *aeruginosa* research in Bangladesh has concentrated on antibiotic resistance profiles in clinical and wastewater isolates, but a thorough examination of environmental isolates in the specified region is lacking. Thus, this study aimed to provide significant insights into the prevalence, antimicrobial resistance, and virulence profiles of multidrug-resistant *P*. *aeruginosa* isolates from clinical, environmental and poultry sources in some selected areas of Bangladesh. The findings of this study can significantly contribute to our knowledge of the dynamics of *P*. *aeruginosa*, which in turn can inform public health policies, clinical practices, and future research efforts aimed at controlling infections caused by this pathogen.

## 2. Materials and methods

### 2.1 Isolation and identification of *P*. *aeruginosa*

This cross-sectional study was conducted in the Bacteriology Laboratory of the Department of Microbiology and Hygiene, Bangladesh Agricultural University (BAU), Mymensingh, Bangladesh during November 2022 to July 2023. The studied samples (**S1 Table in S1 File**) were collected from five different locations including BAU residential area (BAURA), BAU Healthcare Center (BAUHCC), BAU Veterinary Teaching Hospital (BAUVTH), Poultry Market at BAU Sheshmor (PM), and Mymensingh Medical College Hospital (MMCH). A total of 110 samples (46 sources) from the study locations including BAURA = 15, BAUHCC = 20, BAUVTH = 22, PM = 30 and MMCH = 23 were collected (**S1 Table in S1 File**). Therefore, these samples belonged to three major categories including non-hospital environmental samples (BAURA; 15 samples), hospital-based clinical samples (BAUHCC, BAUVTH and MMHC; 65 samples) and poultry samples (PM, 30 samples). These samples comprising drain water, sewage, soil, and samples from hospital environments and poultry markets (**S1 Table in S1 File**), were individually placed in sterile test tubes with 3–4 mL nutrient broth, labelled and transported to the laboratory. A single loopful of overnight culture grown in nutrient broth was streaked over cetrimide agar (HiMedia, India) and aerobically incubated overnight at 37°C. Colonies with distinguishing characteristics, such as greenish color, were assumed to be *Pseudomonas* genus (n = 89 isolates). The genus-specific identification of *P*. *aeruginosa* typically involved a series of standard bacteriological methods including gram staining and an array of biochemical assays, including sugar fermentation, Voges-Proskauer, indole, and catalase tests [4, 19].

### 2.2. Molecular identification of *P*. *aeruginosa*

*Pseudomonas* isolates (n = 89) were then molecularly identified using genus- and species-specific polymerase chain reaction (PCR) assays. Genomic DNA from overnight culture by boiled DNA extraction method using commercial DNA extraction kit, QIAamp DNA Mini Kit (QIAGEN, Hilden, Germany). Quality and quantity of the extracted DNA were measured using a NanoDrop ND-2000 spectrophotometer (Thermo Fisher Scientific, Waltham, MA). DNA extracts with A260/280 and A260/230 ratios of ∼ 1.80 and 2.00 to 2.20, respectively, were considered as high-purity DNA samples [20] and stored at -20°C prior to PCR amplification [21, 22]. To amplify the 16S rRNA sequences of *Pseudomonas*, a set previously designed primer set was used (**Table 1**). PCR amplification of *16s_ Pseudo* for the detection of *Pseudomonas* genus [23] and *Pseudo_aeru* for the detection of *P*. *aeruginosa* species [24, 25] was performed on all phenotypically identified isolates of *P*. *aeruginosa*. The PCR condition against these primer sets for the amplification of genus- and species-specific primers is shown in **S2 Table in S1 File**. Amplification of targeted DNA was carried out in a 20 μL reaction mixture, including 3 μL nuclease-free water, 10 μL 2X master mixture (Promega, Madison, WI, USA), 1 μL forward and reverse primers (for each) and 5 μL DNA template. PCR-positive controls consisted of *P*. *aeruginosa* genomic DNA previously confirmed for the target genes [23–25]. PCR-negative controls utilized non-template controls with PBS instead of genomic DNA. Finally, amplified PCR products underwent electrophoresis on a 1.5% agarose gel and visualized using an ultraviolet transilluminator (Biometra, Gottingen, Germany). A 100 bp DNA ladder (Promega, Madison, WI, USA) was used to validate the expected sizes of the amplified PCR products [26]. Finally, 22 isolates were confirmed as *P*. *aeruginosa* through species-specific PCR.

**Table 1:** Primer sequences used in this study.

Primer	Sequence (5´-3´)	Size (bp)	References
*16s_ Pseudo*	F: GACGGGTGAGTAATGCCTA	618	[23]
	R: CACTGGTGTTCCTTCCTATA		
*16s_Pseuaeru*	F: GGGGGATCTTCGGACCTCA	956	[24, 25]
	R: TCCTTAGAGTGCCCACCCG		
*exoA*	F: GACAACGCCCTCAGCATCACCAGC	396	[53]
	R: CGCTGGCCCATTCGCTCCAGCGCT		
*lasB*	F: TTCTACCCGAAGGACTGATAC	153	[54, 55]
	R: AACACCCATGATCGCAAC		
*algD*	F: TTCCCTCGCAGAGAAAACATC	520	[56]
	R: CCTGGTTGATCAGGTCGATCT		
*blaTEM*	F: CATTTCCGTGTCGCCCTTAT	793	[57]
	R: TCCATAGTTGCCTGACTCCC		
*blaOXA*	F: ACACAATACATATCAACTTCGC	813	[57]
	R: AGTGTGTTTAGAATGGTGATC		
*blaSHV*	F: GGGTTATTCTTATTTGTCGC	615	[57]
	R: TTAGCGTTGCCAGTGCTC		
*blaCMY*	F: CTGCTGCTGACAGCCTCTTT	462	[37]
	R: TTTTCAAGAATGCGCCAGGC		

### 2.3. Antimicrobial susceptibility

The Kirby-Bauer agar disk diffusion method was used to test the susceptibility of the confirmed *P*. *aeruginosa* isolates to different antibiotics in accordance with the guidelines recommended by the Clinical and Laboratory Standards Institute (CLSI, 2021) to determine the sensitivity or susceptibility [27]. The antibiotic disks (Liofilchem^®^, Italy) used in this study included Ciprofloxacin (CIP, 5 g/disc), Gentamicin (GEN, 10 g/disc), Levofloxacin (LEV, 5 g/disc), Penicillin-G (P, 10 g/disc), Aztreonam (ATM, 30 g/disc), Imipenem (IMP, 10 g/disc), Amikacin (AK, 30 g/disc), Ceftazidime (CAZ, 30 g/disc), Doripenem (DOR, 10 g/disc), and Cefepime (CPM, 0 g/disc). Multidrug-resistant (MDR) isolates were those that were resistant to at least three antibiotic classes [28, 29]. The Multiple Antibiotic Resistance (MAR) index was derived by dividing the number of antibiotics an isolate resisted by the total number of antibiotics utilized [30]. *E*. *coli* strain ATCC25922 was used as the negative control in the antimicrobial susceptibility tests.

### 2.4. Molecular detection of antibiotic resistant and virulence genes in *P*. *aeruginosa*

For the detection of the antibiotic resistant and virulence genes in the *P*. *aeruginosa* isolates, simplex PCR assays for beta-lactams resistant genes (e.g., *blaTEM*, *blaCMY*, *blaSHV*, *blaOXA*), and virulent genes including *exoA*, *exoS*, *lasB*, and *algD* were performed with specific primers (**Table 1**). The PCR protocols utilized for detecting these genes were consistent with those described earlier in Section 2.2. Although, a positive control was not used in the PCR for resistance and virulence genes, non-template control (NTC, no template DNA) was used as a negative control.

### 2.5. Statistical analysis

Data were entered into Microsoft Excel 2020^®^ (Microsoft Corp., Redmond, WA, USA) and analyzed using Excel and SPSS version 20 (IBM Corp., Armonk, NY, USA). The Pearson’s chi-square test was performed to compare the prevalence of *P*. *aeruginosa* in three different sample categories (e.g., clinical, environment and poultry). A prevalence percentage was calculated by dividing the number of positive samples for the given category by the total samples tested within that category [31, 32]. The prevalence formula was applied for determining prevalence percentage of *Pseudomonas* and *P*. *aeruginosa*. The AMR patterns, resistance, intermediate and sensitivity, and MAR index were calculated using the CLSI (2021) guideline using the cut-off as provided in the brochure of the manufacturer (Liofilchem^®^, Italy). Correlation coefficients for any of the two resistant antibiotics, association between phenotypic and genotypic resistance patterns, and phenotypic/genotypic resistance patterns and virulence genes of isolated *P*. *aeruginosa* was calculated using Pearson correlation tests. For the test, p < 0.05 was considered statistically significant.

## 3. Results

### 3.1 Prevalence of *P*. *aeruginosa*

From a total of 110 samples collected, 89 isolates were suspected to be *Pseudomonas* genus (80.90%) through culture and biochemical tests. However, the species-specific PCR confirmed 20% (22/110; 95% CI, 13.59–28.43) prevalence of *P*. *aeruginosa* in the studied samples. Non-hospital environment (from BAURA), hospital-based clinical (from BAUHCC, BAUVTH and MMCH) and poultry (PM) samples were found to harbor higher number of *P*. *aeruginosa*. The prevalence of *P*. *aeruginosa* was found to be the highest in the PM (30%, 95% CI, 12.6–47.4) followed by BAURA (26.67%, 95% CI, 12.4–27.6) and MMCH (13.85%, 95% CI, 0.5–22.5). Bivariate analysis exhibited that the hospital-based clinical (BAUHCC, BAUVTH and MMCH) samples had a significant correlation with both non-hospital environment samples and poultry market (PM) samples (Pearson correlation coefficient, ρ = 1; *p* <0.001) (**S3 Table in S1 File**).

### 3.2 Antibiogram profile of *P*. *aeruginosa*

**Fig 1:**
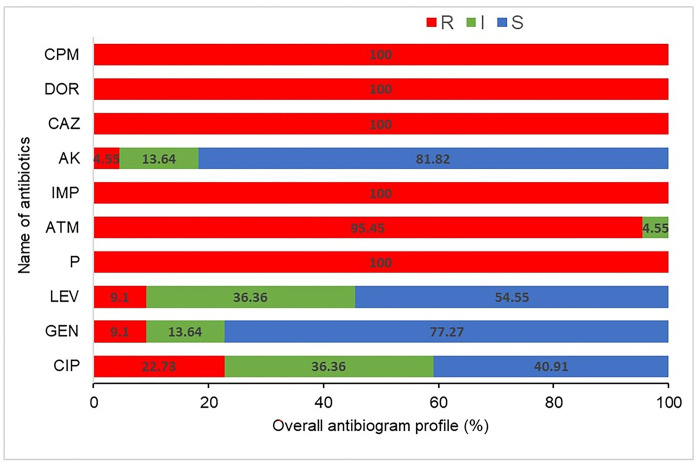
Overall resistance rates of the 22 *P*. *aeruginosa* isolates to 10 antibiotics. Percentage of R (Resistant, red), I (Intermediate resistant), and S (Susceptible) profiles are indicated for each antibiotic inside the bar chart. CIP: Ciprofloxacin, GEN: Gentamicin, LEV: Levofloxacin, P: Penicillin G, ATM: Aztreonam, IMP: Imipenem, AK: Amikacin, CAZ: Ceftazidime, DOR: Doripenem and CPM: Cefepime.

The overall antibiogram profiles of isolated *P*. *aeruginosa* are presented in **Fig 1**. All of the 22 *P*. *aeruginosa* isolates exhibited a concerning trend of antibiotic resistance. Notably, all isolates displayed 100% (95% CI, 85.13–100) resistance to Doripenem, Penicillin, Ceftazidime, Cefepime, and Imipenem, whereas resistance to Aztreonam was observed in 95.45% (95% CI, 78.20–99.76) of the isolates (**Fig 1**). However, variable sensitivity was found against Gentamicin (77.27%, 95% CI, 56.56–89.87), and Levofloxacin (54.55%, 95% CI, 34.66–73.08), Amikacin (81.82%,95% CI, 61.48–92.69), and Ciprofloxacin (40.91%,95% CI, 23.25–61.26) (**Fig 1**). The bivariate analysis demonstrated highly significant correlation between the antibiotic resistant isolates. A high positive significant correlation existed between resistance patterns of Levofloxacin and Ciprofloxacin (ρ = 0.641; *p* = 0.001), Amikacin and Gentamicin (ρ = 0.615; *p* = 0.002) and Levofloxacin and Gentamicin (ρ = 0.569; *p* = 0.006) (**Table 2**). Overall, MDR rate of the *P*. *aeruginosa* isolates was 100%. Among tested antibiotics, seven antibiotic resistance patterns were observed among the isolated *P*. *aeruginosa* isolates. Resistance pattern CAZ, CPM, IMP, DOR, ATM, P (Pattern No. 1) were the most observed (63.64%) among *P*. *aeruginosa* isolates. The MAR indices vary between 0.5 to 0.9 (**Table 3**).

**Table 2:** Comparison of Pearson correlation coefficients between any of two antibiotics resistant to *P*. *aeruginosa*.

		CIP	GEN	LEV	ATM	AK
CIP	Pearson Correlation	1				
	Sig. (2-tailed)					
GEN	Pearson Correlation	.396	1			
	Sig. (2-tailed)	.068				
LEV	Pearson Correlation	.641**	.569**	1		
	Sig. (2-tailed)	.001	.006			
ATM	Pearson Correlation	.230	.110	.182	1	
	Sig. (2-tailed)	.303	.626	.419		
AK	Pearson Correlation	.330	.615**	.171	.096	1
	Sig. (2-tailed)	.134	.002	.447	.671	

A *p*-value less than 0.05 (p<0.05) was considered as significant

**, Correlation is significant at the 0.01 level (2-tailed)

*, Correlation is significant at the 0.05 level (2-tailed). CIP: Ciprofloxacin, GEN: Gentamicin, LEV: Levofloxacin, ATM: Aztreonam, and AK: Amikacin.

**Table 3:** Multidrug-resistance patterns of *P*. *aeruginosa* isolates.

Pattern No.	Antibiotic Resistance Patterns	No. of Antibiotics (Classes)	No. of MDR Isolates (%)	Overall No. of MDR Isolates (%)	MAR indices
1	CAZ, CPM, IMP, DOR, ATM, P	6 (4)	14 (63.64)	22 (100)	0.6
2	CAZ, CPM, IMP, DOR, ATM, P, LEV	7 (5)	1 (4.55)		0.7
3	CIP, CAZ, CPM, IMP, DOR, ATM, P, LEV	8 (5)	1 (4.55)		0.8
4	CIP, CAZ, CPM, IMP, DOR, ATM, P	7 (5)	3 (13.64)		0.7
5	CIP, CAZ, GEN, CPM, IMP, DOR, ATM, P, LEV	9 (6)	1 (4.54)		0.9
6	CAZ, CPM, IMP, DOR, P	5 (3)	1 (4.54)		0.5
7	CAZ, GEN, CPM, IMP, DOR, ATM, AK, P	8 (5)	1 (4.54)		0.8

CIP: Ciprofloxacin, GEN: Gentamicin, LEV: Levofloxacin, P: Penicillin-G, ATM: Aztreonam, IMP: Imipenem, AK: Amikacin, CAZ: Ceftazidime, DOR: Doripenem and CPM: Cefepime.

### 3.3. Antibiotic resistance genes of *P*. *aeruginosa*

**Fig 2:**
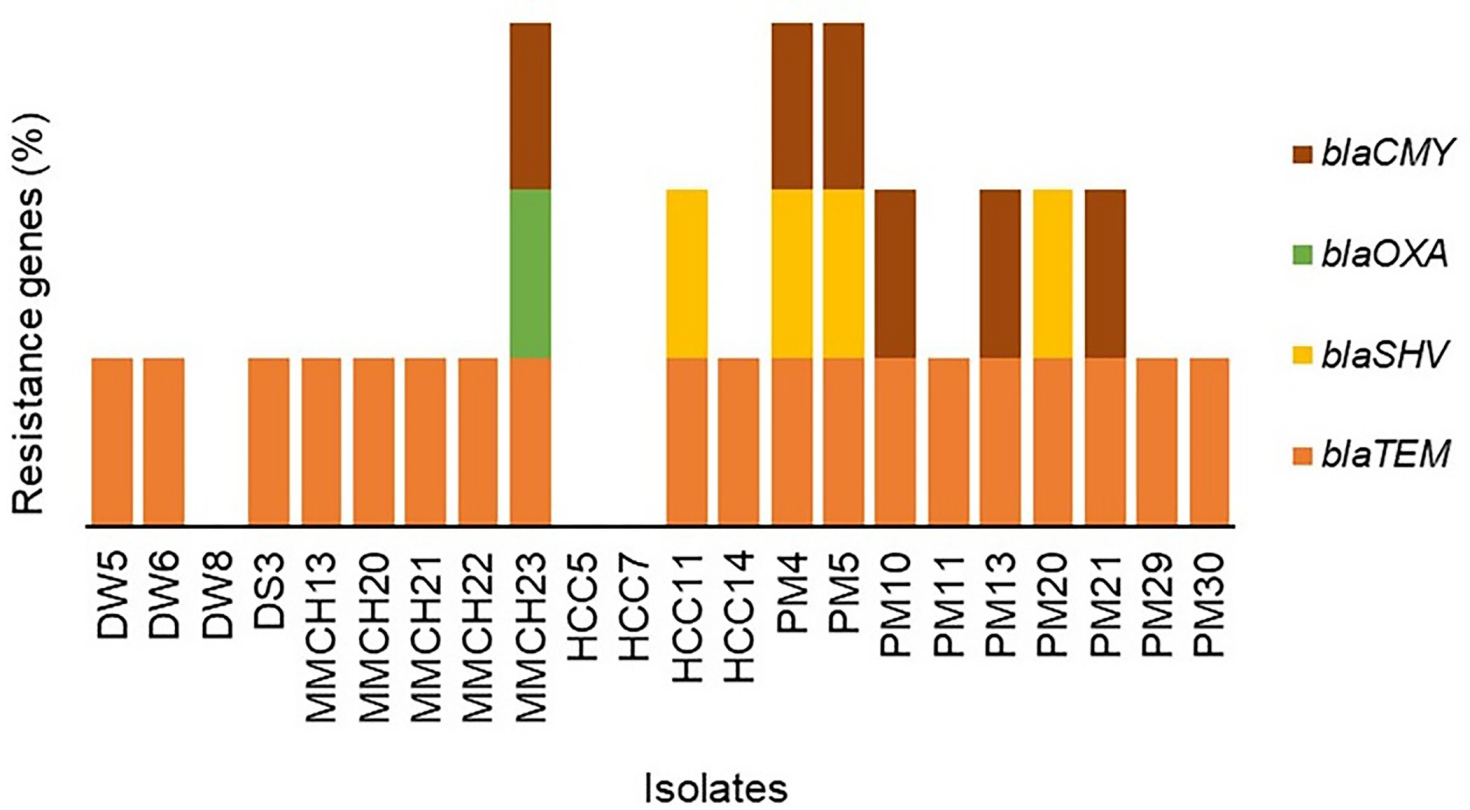
Distribution of antimicrobial resistance (β-lactamase) genes (ARGs) in *P*. *aeruginosa* currently circulating in clinical, environmental and poultry sources in Bangladesh.

The amplified PCR products for **r**esistance-associated *blaTEM*, *blaOXA*, *blaSHV* and *blaCMY* genes in *P*. *aeruginosa* isolates are depicted in **S1 Fig in S1 File** Of the tested isolates (n = 22), 19 (86.36%, 95% CI, 66.66–95.25); 6 (27.27%, 95% CI, 13.15–48.15); 4 (18.18%, 95% CI, 7.30–38.51); and 1 (4.55%, 95% CI, 0.23–21.79) were positive for *blaTEM*, *blaCMY*, *blaSHV*, and *blaOXA*, respectively, which are responsible for beta-lactam antibiotics resistance in *P*. *aeruginosa* (**S1 Fig in S1 File**). Among the resistance genes harboring isolates, 9.09% (95% CI, 1.61–27.81) isolates were positive for the *blaTEM*, *blaSHV*, and *blaOXA* genes; 4.54% (95% CI, 0.23–21.79) were for *blaTEM*, *blaCMY*, and *blaOXA* genes; 9.09% (95% CI, 1.61–27.81) for the *blaTEM*, *blaCMY*, *blaSHV*, and *blaOXA* genes; 13.63% (95% CI, 4.74–33.33) for the *blaTEM* and *blaCMY* genes; and 50% (95% CI, 30.72–69.27) were found to carry a single resistance gene, *blaTEM* (**Fig 2**). By comparing the phenotypic and genotypic resistance pattern of the *P*. *aeruginosa* isolates, we found that the Levofloxacin and Gentamycin resistant isolates had significantly higher amount of *blaCMY* and *blaOXA* than the other two checked genes (*blaTEM* and *blaSHV*) (**Table 4**).

**Table 4:** Association between phenotypic and genotypic resistance patterns of isolated *P*. *aeruginosa*.

Resistance	Genotypic					
	Antibiotics	No (%) of *blaTEM* (n = 19)	No (%) of *blaCMY* (n = 6)	No (%) of *blaSHV* (n = 4)	No (%) of *blaOXA* (n = 1)	*p-*value
Phenotypic	CPM	19 (100^a^)	6 (100^a^)	4 (100^a^)	1 (100^a^)	NA
	DOR	19 (100^a^)	6 (100^a^)	4 (100^a^)	1 (100^a^)	NA
	CAZ	19 (100^a^)	6 (100^a^)	4 (100^a^)	1 (100^a^)	NA
	AK	1(5.3^a^)	0(0^a^)	0(0^a^)	0(0^a^)	0.897
	IMP	19(100^a^)	6(100^a^)	4(100^a^)	1(100^a^)	NA
	ATM	18(94.7^a^)	6	3(75^a^)	1	0.437
	P	19(100^a^)	6(100^a^)	4(100^a^)	1(100^a^)	NA
	LEV	1(5.3^a^)	1(16.7^b^)	0(0^a^)	1(100^b^)	0.017
	GEN	2(10.5^a^)	1(16.7^b^)	0(0^a^)	1(100^b^)	0.050
	CIP	4(21.1^a^)	2(33.3^a^)	1(25^a^)	1(100^a^)	0.362

Each subscript letter denotes a subset of genes categories whose column proportions do not differ significantly from each other at the 0.05 level. CIP: Ciprofloxacin, GEN: Gentamicin, LEV: Levofloxacin, P: Penicillin-G, ATM: Aztreonam, IMP: Imipenem, AK: Amikacin, CAZ: Ceftazidime, DOR: Doripenem and CPM: Cefepime. NA: Not applicable.

### 3.4. Virulence genes in *P*. *aeruginosa*

**Fig 3:**
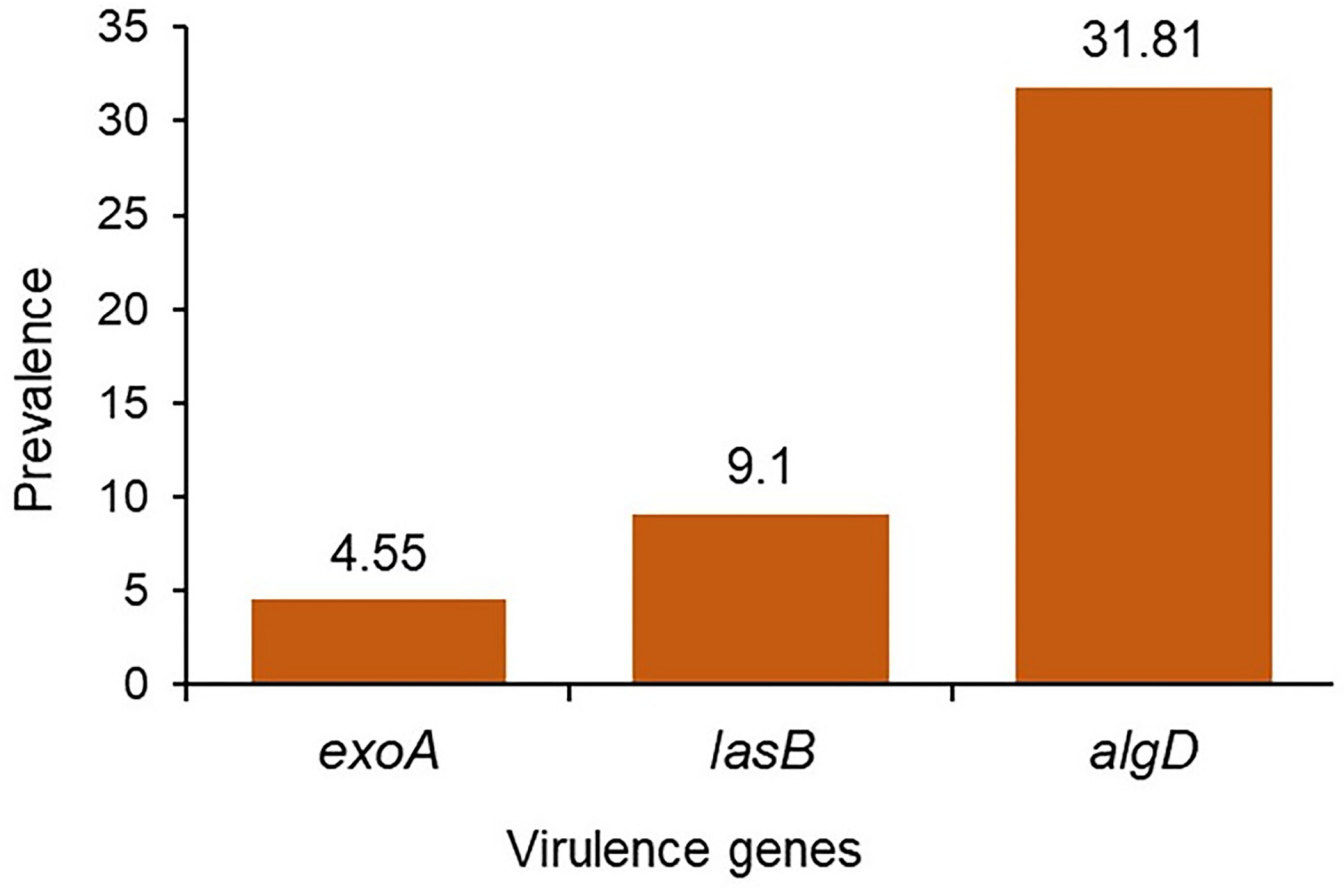
Prevalence of virulence genes in *P*. *aeruginosa* currently circulating in clinical, environmental and poultry sources in Bangladesh.

Gene-specific simplex PCR was used to screen the virulence genes in 22 *P*. *aeruginosa* isolates. Among these isolates, 31.81% (7/22, 95% CI, 16.36–52.68) were positive for the *algD* gene and 9.1% (2/22, 95% CI, 1.61–27.81) were positive for *lasB* gene. However, *exoA* gene was detected only in 4.55% (1/22, 95% CI, 0.23–21.79) *P*. *aeruginosa* isolates (**Fig 3**), while *exoS* gene was absent in the study isolates. Moreover, 9.1% (2/22, 95% CI, 1.61–27.81) isolates were found to carry both *lasB* and *algD* genes, whereas 4.54% (1/22, 95% CI, 0.23–21.79) isolates harbored both *algD* and *exoA* genes. Additionally, by analyzing the association between resistance patterns (phenotypic or genotypic) and virulence profile (genotypic) of the *P*. *aeruginosa* isolates, we found no significant association (**S4 and S5 Tables in S1 File**).

## 4. Discussion

*P*. *aeruginosa* is considered a significant cause of nosocomial or healthcare-associated infections (HAI) and is classified as a priority critical pathogen of global concern by various health organizations [33]. As an opportunistic bacterial pathogen, *P*. *aeruginosa* has been linked with a range of human and animal infections [34]. *P*. *aeruginosa* is ubiquitous in the environment (soil and water) and capable to produce disease in individuals with weakened or compromised immune systems [35]. Despite breakthroughs in medical and surgical treatment, as well as the introduction of a wide range of antimicrobial drugs in livestock and agricultural, the number of resistant isolates in outdoor surface water has increased considerably [36]. Reports from different countries are available describing the detection of *P*. *aeruginosa* from various environmental sources [4, 5, 35, 37]. However, sufficient information is unavailable on the environmental distribution of potential pathogenic MDR *P*. *aeruginosa* in Bangladesh. Previous studies in Bangladesh have focused on clinical isolates, healthcare workers’ mobile phones, and even the genomic diversity of MDR *P*. *aeruginosa* from burn patients [4, 13, 38]. Therefore, this study was designed to determine the antibiotic resistance profiles and virulence genes distribution in *P*. *aeruginosa* circulating in different environments of Bangladesh. We analyzed 110 samples belonged to three major categories e.g., non-hospital environment (BAURA), hospital-based clinical (BAUHCC, BAUVTH and MMHC) and poultry market (PM) from five distinct locations of Mymensingh division of Bangladesh. These diverse study areas collectively provided a comprehensive overview of the prevalence and distribution of MDR *P*. *aeruginosa* and its virulence profiles within distinct environmental contexts, contributing to a holistic understanding of the potential public health significance of these bacteria in the selected region. The overall prevalence of *P*. *aeruginosa* was 20.0%. However, based on locations and sample categories, 26.67%, 13.85%, and 30.0% were reported in non-hospital environment samples (drain water and drain sewages) of BAURA, clinical samples (BAUHCC, BAUVTH and MMCH) and poultry (PM) samples, respectively. The highest isolation rates of *P*. *aeruginosa* (30.0%) was found in the PM samples. In Bangladesh, the average isolation rate for *P*. *aeruginosa* from diverse surface waters was reported to be 61.5%, which is greater than in its neighboring nation, India (45.45%) [39]. The isolation rate of various water samples may fluctuate depending on the source and size of the sample, as well as the time and length of sample collection [3, 37, 40]. Previously, Algammal et al. reported 28.3% prevalence of *P*. *aeruginosa* in broiler chickens in Egypt [41] whereas El-Tawab et al. found that 34% of the tested frozen fish possessed *P*. *aeruginosa* [42]. Detection of *P*. *aeruginosa* in poultry and associated environment is alarming because of the possibilities of transmission of these pathogens to human.

In this study, the most commonly used antibacterial drugs in Bangladesh were tested against *P*. *aeruginosa* isolates to determine their resistance pattern. We found that 100% *P*. *aeruginosa* isolates were resistant to multiple antibiotics such as Penicillin, Ceftazidime, Cefepime, Doripenem and Imipenem. In addition, 95.45% isolates were resistant to Aztreonam. However, the study isolates showed high to moderate level (44.0 to 78.0%) sensitivity against Amikacin, Gentamicin and Ciprofloxacin. These results corroborated with several earlier studies that investigated antimicrobial susceptibility profile in P. aeruginosa isolated from clinical samples [4], environmental samples [38] and poultry samples [43]. Consistent findings across various studies examining the antimicrobial susceptibility profile of *P*. *aeruginosa* isolated from diverse sources (clinical, environmental, and animal samples) can lend support to the understanding of the AMR patterns and prevalence of MDR strains within this bacterium. Such cumulative evidence aids in comprehending the overall resistance landscape and guides the development of effective strategies for managing infections caused by *P*. *aeruginosa*. We found that 100.0% isolates of the *P*. *aeruginosa* were MDR isolates (resistant to > 5 antibiotics). Furthermore, the multiple antibiotic resistance (MAR) indices for the *P*. *aeruginosa* isolates ranged from 0.5 to 0.9 indicating an increased likelihood of these isolates being exposed to various antibiotics or antimicrobial agents, potentially contributing to the development of MDR, posing significant concerns for public health due to the challenges in treating infections caused by these organisms. Indeed, a MAR index greater than 0.2 is indicative of potential contamination from sources where antibiotics are frequently utilized [44].

We also investigated the occurrence of several ARGs which could aid in designing effective treatment strategies and surveillance programs to control the spread of antibiotic-resistant strains of *P*. *aeruginosa*. In this study, *blaTEM* gene was found to be predominating in 86.36% isolates. This high frequency of *blaTEM* is however, consistent with the findings of Hosu et al. who reported a 79.3% prevalence of *blaTEM* in clinical *P*. *aeruginosa* isolates, indicating its widespread dispersion in healthcare settings [11, 45]. the second most prevalent ARG was *blaCMY* which was detected in about 27.0% isolates. Recently, Ejikeugwu et al. reported that around 42.0% *P*. *aeruginosa* isolates from clinical samples harbored *blaCMY* gene [46], which is a bit higher than our findings. This observation is incongruent with the findings of Mohamed et al. who detected a far higher incidence of *blaCMY* in *P*. *aeruginosa* isolates derived from chickens, indicating possible sources of resistance in non-clinical settings [47]. Although, *blaSHV*, and *blaOXA* were identified as the less abundant ARG in *P*. *aeruginosa* isolates (18.0% and 4.55%, respectively), these ARGs were found in different combination. The detection of *blaSHV*, and *blaOXA* genes in *P*. *aeruginosa* isolates aligns with findings from previous studies [4, 48, 49], further highlighting the significance of these genes in healthcare settings and their association with nosocomial infections. Their presence underscores the potential for these bacteria to cause severe infections and emphasizes the need for stringent infection control measures and judicious use of antibiotics to mitigate the emergence and spread of multidrug-resistant strains The difference between the results of this study and other investigations might be attributed to differences in various circumstances comprising incubators or to commonly occurring hyper-mutation among *P*. *aeruginosa* strains exhibiting varied antibiotic resistance. Furthermore, antibiotic-resistant bacteria can swiftly spread across the food chain and cause the majority of public health problems [13, 36].

One of the hallmark findings of this study is the identification several virulence genes in the *P*. *aeruginosa* isolates that might contribute to the pathogenicity of this bacterium in multiple hosts. The presence of the *algD* and *lasB* genes in around 32.0% and 9.0% of the *P*. *aeruginosa* isolates, respectively, suggests the potential virulence of these strains. The *algD* gene is associated with alginate production, which contributes to the formation of biofilms and enhances the bacteria’s resistance to host immune responses and antibiotics [50, 51]. The *lasB* gene encodes for elastase, an enzyme involved in tissue damage and immune evasion [52]. On the other hand, the low prevalence of the *exoA* gene (4.55%) and the absence of the *exoS* gene indicate the variability in the distribution of virulence factors among these isolates. The *exoA* gene encodes exotoxin A, a cytotoxin known to interfere with host cell function, contributing to cytotoxicity and the establishment of infections [53]. Therefore, the differential presence and absence of these virulence genes among the isolates underscore the heterogeneity of *P*. *aeruginosa* strains and their potential variation in virulence and pathogenicity.

## 5. Conclusions

To the best of our knowledge, this is the first instance of reporting the presence of multidrug-resistant *P*. *aeruginosa* in diverse environmental samples, including non-hospital residential areas (BAURA), clinical settings in both human and animal hospitals, and samples from poultry markets in Bangladesh. We found that *P*. *aeruginosa* is prevalent in these diverse group of samples at 20.0%, and highest prevalence was recorded in poultry samples (30.0%). Despite having discrepancy in prevalence according to sample types, 100.0% *P*. *aeruginosa* isolates showed resistance against at least five antibiotics tested. Additionally, identified MDR *P*. *aeruginosa* were found to harbor beta-lactamase genes such as *blaTEM*, *blaCMY*, *blaSHV*, and *blaOXA* and virulence genes including *exoA*, *lasB*, and *algD*.

The occurence of MDR *P*. *aeruginosa* is highly concerning, as both humans and animals can easily acquire these potential pathogens from the environment, leading to infections. It is crucial to implement routine surveillance and consistently practice the regular disinfection of environmental surfaces to effectively diminish their prevalence in various settings. Indeed, there are several promising avenues for further research on the *P*. *aeruginosa* isolates obtained in this study. Further studies with a larger cohort of samples on epidemiological source tracking, strain typing, determining the minimum inhibitory concentration (MIC) of antibiotics, resistome, and pathogenicity profiles will enhance our understanding of *P*. *aeruginosa* infections. Therefore, regular monitoring of AMR and strict adherence to infection control practices would provide the most effective defense against the continual spread of MDR *P*. *aeruginosa* in Bangladesh and beyond.

## Supporting information

S1 File S1 Fig PCR amplification for resistance-associated *blaTEM*, *blaOXA*, *blaSHV* and *blaCMY* gene in *P*. *aeruginosa*. (a) *blaTEM* gene: Lane M = 100 bp DNA ladder, Lane 1–8: positive results of suspected isolates with an amplicon size of 793 bp, and NC: negative control. (b) *blaOXA* gene: Lane M = 100 bp DNA ladder, Lane 1 = positive result of suspected isolates with an amplicon size of 813 bp, and NC: negative control. (c) *blaSHV* gene: Lane M = 100 bp DNA ladder, Lane 1–4: positive result of suspected isolates with an amplicon size of 615 bp, and NC: negative control. (d) *blaCMY* gene: Lane M = 100 bp DNA ladder; Lane 1–6: positive result of suspected isolates with an amplicon size of 462 bp, and NC: negative control. S1 Table. Number of samples used for bacteriological assessment. S2 Table. PCR condition against different primer sequences used for the amplification of *Pseudomonas aeruginosa* isolates, their antimicrobial resistance genes and virulence genes. S3 Table. Comparison of Pearson correlation coefficients among the prevalence of *P*. *aeruginosa* in three different sample categories. S4 Table. Association between phenotypic resistance patterns and virulence genes of isolated *P*. *aeruginosa*. S5 Table. Association between genotypic resistance patterns and virulence of isolated *P*. *aeruginosa*.
(PDF)
