# Supplementary material for: Characterization of β-lactamase and virulence genes in Pseudomonas aeruginosa isolated from clinical, environmental and poultry sources in Bangladesh
Source: PLoS One. 2024 Apr 16;19(4):e0296542. doi: 10.1371/journal.pone.0296542 (PMC11020970; doi:10.1371/journal.pone.0296542)
Supplement: S1 File — S1 Fig PCR amplification for resistance-associated blaTEM, blaOXA, blaSHV and blaCMY gene in P. aeruginosa. (a) blaTEM gene: Lane M = 100 bp DNA ladder, Lane 1–8: positive results of suspected isolates with an amplicon size of 793 bp, and NC: negative control. (b) blaOXA gene: Lane M = 100 bp DNA ladder, Lane 1 = positive result of suspected isolates with an amplicon size of 813 bp, and NC: negative control. (c) blaSHV gene: Lane M = 100 bp DNA ladder, Lane 1–4: positive result of suspected isolates with an amplicon size of 615 bp, and NC: negative control. (d) blaCMY gene: Lane M = 100 bp DNA ladder; Lane 1–6: positive result of suspected isolates with an amplicon size of 462 bp, and NC: negative control. S1 Table. Number of samples used for bacteriological assessment. S2 Table. PCR condition against different primer sequences used for the amplification of Pseudomonas aeruginosa isolates, their antimicrobial resistance genes and virulence genes. S3 Table. Comparison of Pearson correlation coefficients among the prevalence of P. aeruginosa in three different sample categories. S4 Table. Association between phenotypic resistance patterns and virulence genes of isolated P. aeruginosa. S5 Table. Association between genotypic resistance patterns and virulence of isolated P. aeruginosa. (PDF) [file pone.0296542.s001.pdf]

Supporting Figure

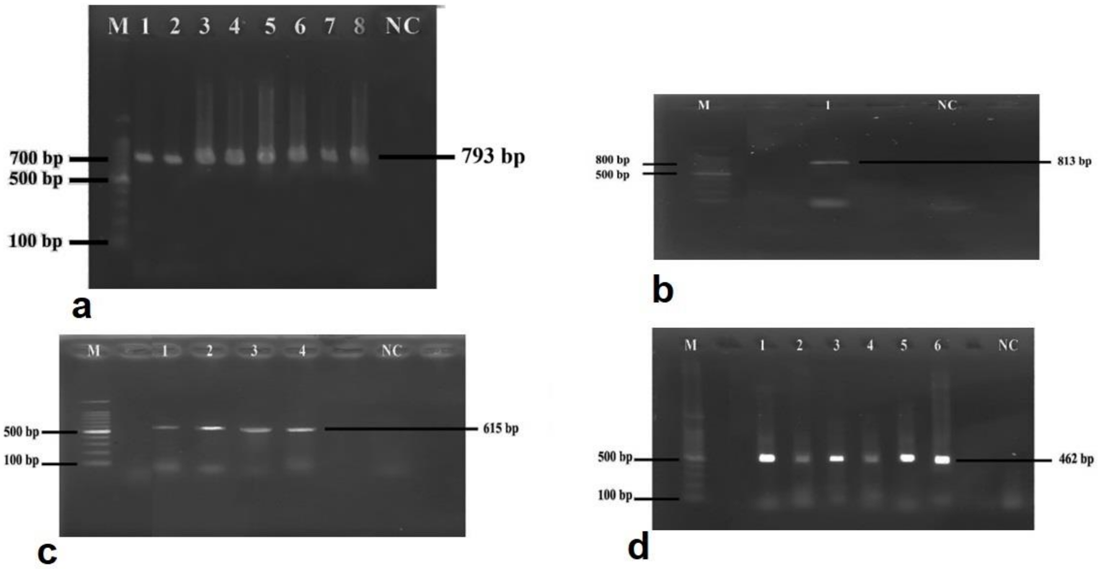

Fig. S1: PCR amplification for **r**esistance-associated *blaTEM*, *blaOXA*, *blaSHV* and *blaCMY* gene in *P. aeruginosa*. (a) *blaTEM* gene: Lane M=100 bp DNA ladder, Lane 1-8: positive results of suspected isolates with an amplicon size of 793 bp, and NC: negative control. (b) *blaOXA* gene: Lane M=100 bp DNA ladder, Lane 1= positive result of suspected isolates with an amplicon size of 813 bp, and NC: negative control. (c) *blaSHV* gene: Lane M=100 bp DNA ladder, Lane 1-4: positive result of suspected isolates with an amplicon size of 615 bp, and NC: negative control. (d) *blaCMY* gene: Lane M=100 bp DNA ladder; Lane 1-6: positive result of suspected isolates with an amplicon size of 462 bp, and NC: negative control.

Supporting Tables

Table S1: Number of samples used for bacteriological assessment

Sources	No. of samples types	Samples type	No. of samples	Total
BAURA	2	Drain water<br>Drain sewages	8+7	15
MMCH	14	Nurse Desk<br>Different room floor<br>Hospital trolley<br>Saline stand<br>Door samples<br>Bed<br>Switch<br>Wheel Chair handle<br>Cleaning stuff<br>Washroom basins<br>Stair railing<br>Canteen basins<br>Canteen table<br>Hospital area swabs	1+4+1+1+1+3+2+1+<br>1+2+1+1+1+3	23
BAUHCC	13	Floor<br>Bed<br>Bed steel<br>Hospital tray<br>Oxygen cylinder<br>Chair surface<br>Oxygen holder<br>Switch<br>Door handle<br>Washroom floor<br>Bed surface<br>Door surface<br>Drain swab	2+1+1+1+1+2+1+1+<br>1+3+1+1+4	20
BAUVTH	7	Drain swab<br>Hospital floor<br>Weight machine surface<br>Cowshed drain swabs<br>Waste water<br>Drain water<br>Hospital surface	3+2+2+2+2+5+6	22
BAUPM	10	Cloacal swabs<br>Feces from different floor<br>Feces from feeding area<br>Tracheal swabs<br>Feeder surface<br>Floor with feces<br>Chicken rearing floor	9+6+6+2+1+1+1+1+<br>2+1	30

Sources	No. of samples types	Samples type	No. of samples	Total
		Feeding area surface<br>Feed from feeder<br>Feeder surface with feed		
**Grand Total**	**46**			**110**

Here: BAURA: BAU residential area; MMCH: Mymensingh Medical College Hospital; BAUHCC: BAU Health Care Centre; BAUVTH: BAU Veterinary Teaching Hospital, PM: BAU sheshmor poultry market. Hospital sample: MMCH, BAUHCC and BAUVTH, non-hospital Environment samples: BAURA, Poultry samples: PM

Table S2. PCR condition against different primer sequences used for the amplification of *Pseudomonas aeruginosa* isolates, their antimicrobial resistance genes and virulence genes.

**Primer**	**Initial denatu-ration**		**Denaturation**		**Annealing**		**Elongation**		**Final extension**		**References**
	Temp	Time	Temp	Time	Temp	Time	Temp	Time	Temp	Time	
16s_ Pseudo	95°C	3m	95°C	20 s	54°C	20 s	72°C	40 s	72°C	10 m	[1]
Pseudo _aeru	95°C	2m	94°C	20 s	58°C	20 s	72°C	40 s	72°C	10 m	[2, 3]
exoA	95°C	5m	95°C	30s	60°C	30 s	72°C	30 s	72°C	7 m	[4]
lasB	94°C	5m	94°C	30 s	56°C	30 s	72°C	1 m	72°C	12 m	[5, 6]
algD	94°C	5m	94°C	1m	60°C	1 m	72°C	1 m	72°C	7 m	[7]
blaTEM	94°C	3m	94°C	1m	55°C	1 m	72°C	1 m	72°C	10 m	[8]
blaOXA	94°C	5m	94°C	1 m	58°C	1 m	72°C	1 m	72°C	10 m	[8]
blaSHV	95°C	3m	95°C	30s	56°C	40 s	72°C	50 s	72°C	3 m	[8]
blaCMY	94°C	5m	94°C	1 m	64°C	1 m	72°C	1 m	72°C	10 m	[9]

Legend: Temp = Temperature; m = minute; s = second

Reference

1. Spilker, T., et al., *PCR-based assay for differentiation of Pseudomonas aeruginosa from other Pseudomonas species recovered from cystic fibrosis patients.* Journal of clinical microbiology, 2004. **42**(5): p. 2074-2079.
2. RAJA, C.E., R. PANDEESWARİ, and U. RAMESH, *Characterization of high fluoride resistant Pseudomonas aeruginosa species isolated from water samples.* Environmental Research and Technology, 2022. **5**(4): p. 325-339.
3. Anzai, Y., et al., *Phylogenetic affiliation of the pseudomonads based on 16S rRNA sequence.* International journal of systematic and evolutionary microbiology, 2000. **50**(4): p. 1563-1589.
4. Javanmardi, F., et al., *A systematic review and meta-analysis on Exo-toxins prevalence in hospital acquired Pseudomonas aeruginosa isolates.* Infection, Genetics and Evolution, 2019. **75**: p. 104037.
5. Cathcart, G.R., et al., *Novel inhibitors of the Pseudomonas aeruginosa virulence factor LasB: a potential therapeutic approach for the attenuation of virulence mechanisms in pseudomonal infection.* Antimicrobial agents and chemotherapy, 2011. **55**(6): p. 2670-2678.
6. Everett, M.J. and D.T. Davies, *Pseudomonas aeruginosa elastase (LasB) as a therapeutic target.* Drug Discovery Today, 2021. **26**(9): p. 2108-2123.
7. Taee, S.R., et al., *Detection of algD, oprL and exoA genes by new specific primers as an efficient, rapid and accurate procedure for direct diagnosis of Pseudomonas aeruginosa strains in clinical samples.* Jundishapur journal of microbiology, 2014. **7**(10).
8. Bahrami, M., M. Mmohammadi-Sichani, and V. Karbasizadeh, *Prevalence of SHV, TEM, CTX-M and OXA-48 β-Lactamase genes in clinical isolates of Pseudomonas aeruginosa in Bandar-Abbas, Iran.* Avicenna Journal of Clinical Microbiology and Infection, 2018. **5**(4): p. 86-90.
9. Fallah, F., et al., *Evaluating the antimicrobial resistance and frequency of AmpC β-lactamases blaCMY-2 gene in Gram-negative bacteria isolates collected from selected hospitals of Iran: a multicenter retrospective study.* Gene Reports, 2020. **21**: p. 100868.

Table S3. Comparison of Pearson correlation coefficients among the prevalence of *P. aeruginosa* in three different sample categories.

		Non-hospital environmental sample	Hospital-based clinical sample	Poultry market sample
Non-hospital environment Sample	Pearson Correlation	1		
	Sig. (2-tailed)	-		
Hospital-based clinical sample	Pearson Correlation	1.000**	1	
	Sig. (2-tailed)	0.000	-	
Poultry market sample	Pearson Correlation	1.000**		1
	Sig. (2-tailed)	0.000		-

Legends, A *p*-value less than 0.05 was deemed statistically significant; **. Correlation is significant at the 0.01 level (2- tailed); Sig.= Significance.

Table S4. Association between phenotypic resistance patterns and virulence genes of isolated *P. aeruginosa*.

**Resistance**	Antibiotics	**Virulence**			
		No (%) of *exoA* (n=1)	No (%) of *lasB* (n=2)	No (%) of *algD* (n=7)	*p*-value
**Phenotypic**	CPM	1(100[a])	2(100[a])	7(100[a])	NA
	DOR	1(100[a])	2(100[a])	7(100[a])	NA
	CAZ	1(100[a])	2(100[a])	7(100[a])	NA
	AK	0(0[a])	0(0[a])	0(0[a])	NA
	IMP	1(100[a])	2(100[a])	7(100[a])	NA
	ATM	1(100[a])	2(100[a])	7(100[a])	NA
	P	1(100[a])	2(100[a])	7(100[a])	NA
	LEV	0(0[a])	1(50[a])	1(14.3[a])	0.468
	GEN	0 (0[a])	1 (50[a])	1(14.3[a])	0.468
	CIP	0(0[a])	1(50[a])	3(42.9[a])	0.679

Each subscript letter denotes a subset of genes categories whose column proportions do not differ significantly from each other at the 0.05 level. NA: Not applicable.

Table S5. Association between genotypic resistance patterns and virulence of isolated *P. aeruginosa*.

**Resistance**	Resistance genes	Virulence genes			
		No (%) of *exoA* (n=1)	No (%) of *lasB* (n=2)	No (%) of *algD* (n=7)	*p*-value
**Genotype**	bla_{TEM}	1(100[a])	2(100[a])	7(100[a])	NA
	bla_{CMY}	1(100[a])	1(50[a])	2(28.6[a])	0.375
	bla_{SHV}	0(0[a])	0(0[a])	1(14.3[a])	0.788
	bla_{OXA}	0(0[a])	1(50[a])	1(14.3[a])	0.468

Each subscript letter denotes a subset of genes categories whose column proportions do not differ significantly from each other at the 0.05 level. NA: Not applicable.
